# Deep learning-based predictive model for pathological complete response to neoadjuvant chemotherapy in breast cancer from biopsy pathological images: a multicenter study

**DOI:** 10.3389/fphys.2024.1279982

**Published:** 2024-01-31

**Authors:** Huancheng Zeng, Siqi Qiu, Shuxin Zhuang, Xiaolong Wei, Jundong Wu, Ranze Zhang, Kai Chen, Zhiyong Wu, Zhemin Zhuang

**Affiliations:** ^1^ The Breast Center, Cancer Hospital of Shantou University Medical College, Shantou, China; ^2^ Diagnosis and Treatment Center of Breast Diseases, Shantou Central Hospital, Shantou, China; ^3^ Clinical Research Center, Shantou Central Hospital, Shantou, China; ^4^ School of Biomedical Engineering, Sun Yat-sen University, Shenzhen, China; ^5^ The Pathology Department, Cancer Hospital of Shantou University Medical College, Shantou, China; ^6^ Breast Tumor Center, Sun Yat-Sen Memorial Hospital, Guangzhou, China; ^7^ Engineering College, Shantou University, Shantou, China

**Keywords:** breast cancer, deep learning, neoadjuvant chemotherapy, pathological complete response, pathological images

## Abstract

**Introduction:** Early predictive pathological complete response (pCR) is beneficial for optimizing neoadjuvant chemotherapy (NAC) strategies for breast cancer. The hematoxylin and eosin (HE)-stained slices of biopsy tissues contain a large amount of information on tumor epithelial cells and stromal. The fusion of pathological image features and clinicopathological features is expected to build a model to predict pCR of NAC in breast cancer.

**Methods:** We retrospectively collected a total of 440 breast cancer patients from three hospitals who underwent NAC. HE-stained slices of biopsy tissues were scanned to form whole-slide images (WSIs), and pathological images of representative regions of interest (ROI) of each WSI were selected at different magnifications. Based on several different deep learning models, we propose a novel feature extraction method on pathological images with different magnifications. Further, fused with clinicopathological features, a multimodal breast cancer NAC pCR prediction model based on a support vector machine (SVM) classifier was developed and validated with two additional validation cohorts (VCs).

**Results:** Through experimental validation of several different deep learning models, we found that the breast cancer pCR prediction model based on the SVM classifier, which uses the VGG16 model for feature extraction of pathological images at ×20 magnification, has the best prediction efficacy. The area under the curve (AUC) of deep learning pathological model (DPM) were 0.79, 0.73, and 0.71 for TC, VC1, and VC2, respectively, all of which exceeded 0.70. The AUCs of clinical model (CM), a clinical prediction model established by using clinicopathological features, were 0.79 for TC, 0.73 for VC1, and 0.71 for VC2, respectively. The multimodal deep learning clinicopathological model (DPCM) established by fusing pathological images and clinicopathological features improved the AUC of TC from 0.79 to 0.84. The AUC of VC2 improved from 0.71 to 0.78.

**Conclusion:** Our study reveals that pathological images of HE-stained slices of pre-NAC biopsy tissues can be used to build a pCR prediction model. Combining pathological images and clinicopathological features can further enhance the predictive efficacy of the model.

## 1 Introduction

Breast cancer is the most prevalent malignancy worldwide and the leading cause of cancer-related death (Torre et al., 2017). For patients with locally advanced breast cancer or some large operable tumors, neoadjuvant chemotherapy (NAC) is a standard-of-care treatment option (Derks and van de Velde, 2018). According to literature (von Minckwitz et al., 2013), NAC is used to reduce tumor burden and increase breast conservation rates, as well as *in vivo* evaluation of the treatment efficacy of different treatment options. Patients who obtain tumor pathological complete response (pCR) after NAC have a better prognosis than those who do not, also known as non-pCR patients (Cortazar et al., 2014). However, breast cancer is highly heterogeneous and treatment protocols developed by relying solely on molecular typing still have major limitations (Glaeser et al., 2019). Therefore, early and accurate prediction of the efficacy of NAC for breast cancer is important to optimize individualized treatment strategies.

Currently, several clinicopathological features and biomolecular markers, including tumor size (Goorts et al., 2017), histological grading (Alba et al., 2016), Ki67 (Alba et al., 2016), immunochemical (IHC)-based molecular typing (Haque et al., 2018) and stromal tumor-infiltrating lymphocytes (sTILs) are frequently used to predict pCR (Ali et al., 2017; Denkert et al., 2018). However, these simple parameters are not accurate enough to predict NAC efficacy in all breast cancers. Besides, some imaging modalities, such as ultrasound (Jiang et al., 2021), magnetic resonance imaging (MRI) (Cain et al., 2019), and positron emission tomography-computed tomography (PET-CT) (Lee et al., 2019), have been used to predict NAC efficacy, but repeated imaging examinations can lead to additional financial expenses, especially for MRI and PET-CT. Therefore, there is still an urgent need to develop more reliable and inexpensive methods for early prediction of pCR in breast cancer NAC.

Pathological images provide information on various tumor phenotypes and also reflect underlying molecular processes and disease progression, which can provide intrinsic disease information to the clinic. Since human assessment of histological images is mainly based on visual examination by pathologists, the complex and rich information from histological images is difficult to fully utilize. Deep learning (DL) techniques can assist in solving this problem by integrating a large amount of information in complex images (Echle et al., 2021). Recent studies have found that the combination of digital pathology and artificial intelligence (AI) techniques enables the extraction of hidden and quantitative information from histological images, potentially providing information for predicting the therapeutic effect (Acs et al., 2020). In particular, convolutional neural networks (CNN) can efficiently perform difficult visual tasks by learning features from training data (Li et al., 2022b). Currently, DL-based image processing and analysis have been attempted for performing tumor cell identification (Ehteshami Bejnordi et al., 2017), histological grading (Bulten et al., 2020), and immunohistochemical scoring (Akbar et al., 2015), demonstrating considerable application promise. Several studies have shown that it is feasible to develop new biomarkers for predicting anti-tumor treatment efficacy and patient prognosis using medical picture-based DL methods (Beck et al., 2011; Bhargava et al., 2020; Zhang et al., 2020). However, there are fewer studies using hematoxylin and eosin (HE) -stained histological images to predict the efficacy of NAC in breast cancer, a research area that we consider worthy to be explored.

Currently, HE-stained tissue slices can be digitally scanned to form whole-slide images (WSIs). Each WSI contains both tumor cellular and stromal areas that are diagnostically helpful, as well as areas of tumor necrosis and blank areas that may be confusing, and the former are the areas that we can use and need to focus on. In addition, pathologists always need to switch between different magnifications to view pathological images because the combination of different magnification fields provides more comprehensive diagnostic information.

In this multicenter retrospective study, firstly, based on several different deep learning models, a novel feature extraction method on pathological images with different magnifications was proposed. Furthermore, based on the SVM classifier**,** a deep learning pathological model (DPM) to predict NAC pCR in breast cancer was built. In addition, a clinical model (CM) based on clinicopathological features was established. Finally, the prediction efficacy of the multimodal deep learning clinicopathological model (DPCM) was assessed.

## 2 Materials and methods

### 2.1 Patients

**FIGURE 1:**
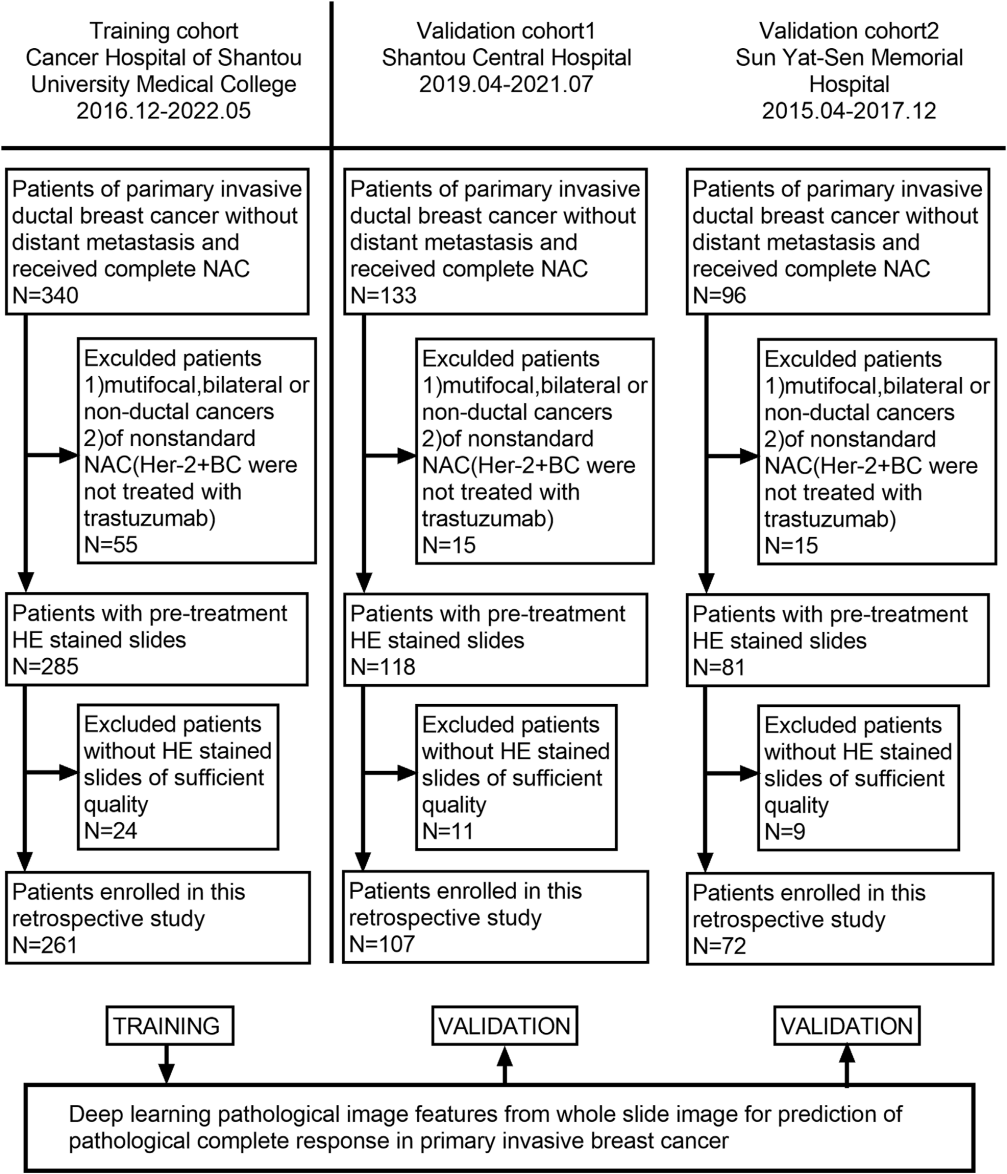
Flowchart of patient enrollment. A total of 440 patients with WSI were enrolled from three hospitals.

Our patients were retrospectively recruited from three hospitals: Cancer Hospital of Shantou University Medical College, Shantou Central Hospital, and Yat-sen Memorial Hospital of Sun Yat-sen University. According to the inclusion and exclusion criteria, 129 patients were excluded from 569 patients, and a total of 440 patients who received NAC between December 2016 and July 2021 were recruited. Among them, 261 patients were enrolled from the Cancer Hospital of Shantou University Medical College, which had the largest number of enrollments and served as the training cohort (TC). Shantou Central Hospital and Yat-sen Memorial Hospital of Sun Yat-sen University enrolled 107 and 72 patients, respectively, as validation cohort 1 (VC1) and validation cohort 2 (VC2). The detailed recruitment flow chart is shown in Figure 1. Patient inclusion criteria were as follows: 1. Female patients with primary invasive ductal carcinoma (IDC) of the breast diagnosed by core needle biopsy; 2. Patients received a complete NAC regimen in parallel with surgical treatment and pathological assessment of NAC efficacy; 3. Patients with digitized HE-stained tissue slices are available. Exclusion criteria were as follows: 1. Patients received non-standard treatment regimens, mainly patients with human epidermal growth factor receptor 2 (Her-2) positive tumors but not treated with trastuzumab; 2. Diagnosis of bilateral or multifocal invasive breast cancer, or invasive breast cancer of special type; 3. Patients with poor quality of WSIs. Our project was approved by the Medical Ethics Committee of Cancer Hospital of Shantou University Medical College (Approval number: 2022125) and followed the Declaration of Helsinki before the tissue samples were used exclusively for scientific research. The medical ethics committee waived the need to obtain informed consent from participants.

### 2.2 NAC pCR prediction model for breast cancer based on multimodal features

**FIGURE 2:**
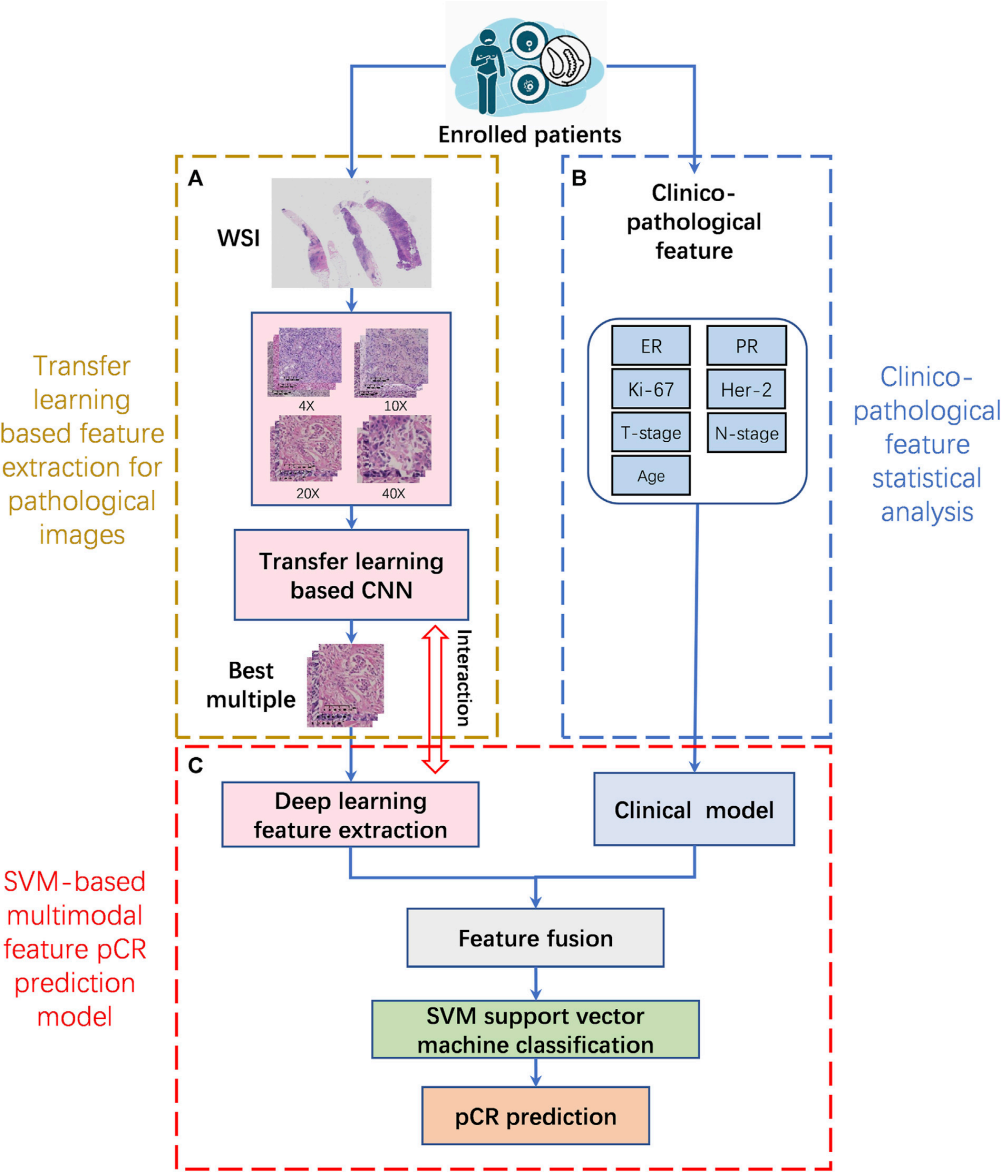
The implementation framework structure of pCR prediction model. **(A)**. Transfer learning based feature extraction for pathological images (TLFEPI Module): ROIs selected from each WSI under different magnifications were feature extracted using transfer learning. **(B)**. Clinicopathological feature extraction (CPFE Module): The clinicopathological features were analyzed by univariate analysis and logistics regression analysis. **(C)**. SVM-based multimodal feature pCR prediction model (SMFPM Module): Feature fusion of pathological images and clinicopathology features, using SVM support vector machine classification to construct a multimodal feature pCR prediction model. pCR: pathologic complete response.

For patients who met the inclusion criteria, the selected ROIs from each WSI at different magnifications were first subjected to feature extraction using a transfer learning model. Then the clinicopathological features were analyzed using statistical methods. Finally, the multimodal feature pCR prediction model is constructed by combining pathological image features and clinicopathological features. The implementation framework structure is shown in Figure 2.

**FIGURE 3:**
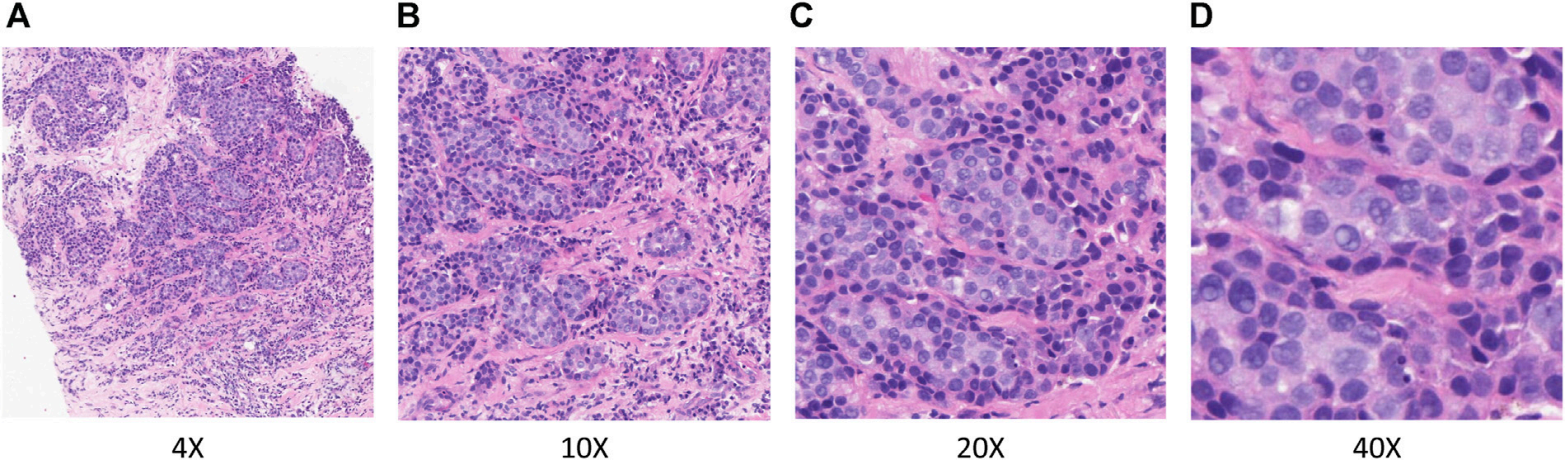
Representative ROIs in different magnifications. **(A)** ×.4 magnification **(B)** ×.10 magnification **(C)** ×.20 magnification **(D)** ×.40 magnification.

In TLFEPI (Transfer learning-based feature extraction for pathological images) Module, the eligible HE-stained tissue slices were digitally scanned at ×40 magnification to form WSIs. Screenshots of five regions of interest (ROI) from each WSI were taken with a fixed screenshot size setting of 512×512 pixels at different magnifications (×4, 10X, 20X, 40X) of the field of the view. The ROIs were selected jointly by a breast surgeon (HCZ) and a pathologist (WLW) with more than 10 years of working experience. Both of these two researchers were unaware of the pCR status of patients. The following criteria were used for ROIs selection: 1. Excluding tumor necrosis area, cell overlap area, blank or margin area; 2. The ROIs need to contain both tumor and stromal areas, with the tumor area accounting for more than 50% of the total area. Representative ROIs with different magnifications are shown in Figure 3.

As mentioned above, each WSI selects 5 ROIs at different magnifications. Taking 4X multiples as an example, a total of 1305 ROIs were selected from 261 patients in TC, of which 75 patients received pCR, and a total of 375 ROIs were selected. The remaining 186 patients received non-pCR, and a total of 930 ROIs were selected. To avoid overfitting due to the small amount of data, we performed data enhancement operations on all ROIs under 4X multiples, such as rotation and inversion, and expanded the number of ROIs to 5790, among which the ROIs for pCR patients was 3000, and that for non-pCR patients was 2790. Using the same method, the ROI under 10X, 20X, and 40X multiples is also expanded to 5790. Therefore, the pathological image data sets under four multiples of 4X, 10X, 20X, and 40X were constructed.

On this basis, we first selected two classification models, VGG16 and ResNet50, as the benchmark models, and trained and tested them on different magnifications and mix magnifications pathological image datasets of TC respectively through the transfer learning. The experimental results show that the ×20 magnification pathological images of TC have the best prediction efficacy in both VGG16 and ResNet50 classification models. Then, we trained and tested TC 20X-multiple pathological images using different series of VGG, ResNet, ResNeSt, and DenseNet models, and the experimental results showed that the VGG16 model had the best prediction efficacy. Therefore, in this study, the VGG16 model is used as an image feature extractor for extracting features from ×20-magnification pathological images to construct a deep learning model (DPM) for predicting pCR of NAC in breast cancer. Then, we validate the prediction efficacy of the DPM using the ROIs selected by VC1 and VC2 at ×20 magnification.

In CPFE (Clinicopathological feature statistical analysis) Module, we collected 7 important clinicopathological indicators, including age at diagnosis, clinical T stage, clinical N stage, estrogen receptor (ER), progesterone receptor (PR), Her-2, and Ki67. ER, PR, Her-2 status and Ki67 expression were assessed by IHC. ER/PR positivity was defined as no less than 1% of tumor cells with positive nuclear staining (Allison et al., 2020). Regarding Ki67, samples were divided into a high expression group (≥20%) and a low expression group (<20%) (Goldhirsch et al., 2013). Her-2 positivity was defined as IHC (3+) and/or amplification by fluorescence *in situ* hybridization (FISH), and Her-2 negativity was defined as IHC (0/1+) and/or non-amplification by FISH(Wolff et al., 2018). In this study, pCR was defined as ypT0/isypN0 (breast and nodes without residual invasive disease) (Cortazar et al., 2014). Through univariate analysis and logistic regression analysis of clinicopathological features of TC, we constructed a clinicopathological features-based prediction model (CM). We validated CM using clinicopathological features of VC1 and VC2.

**FIGURE 4:**
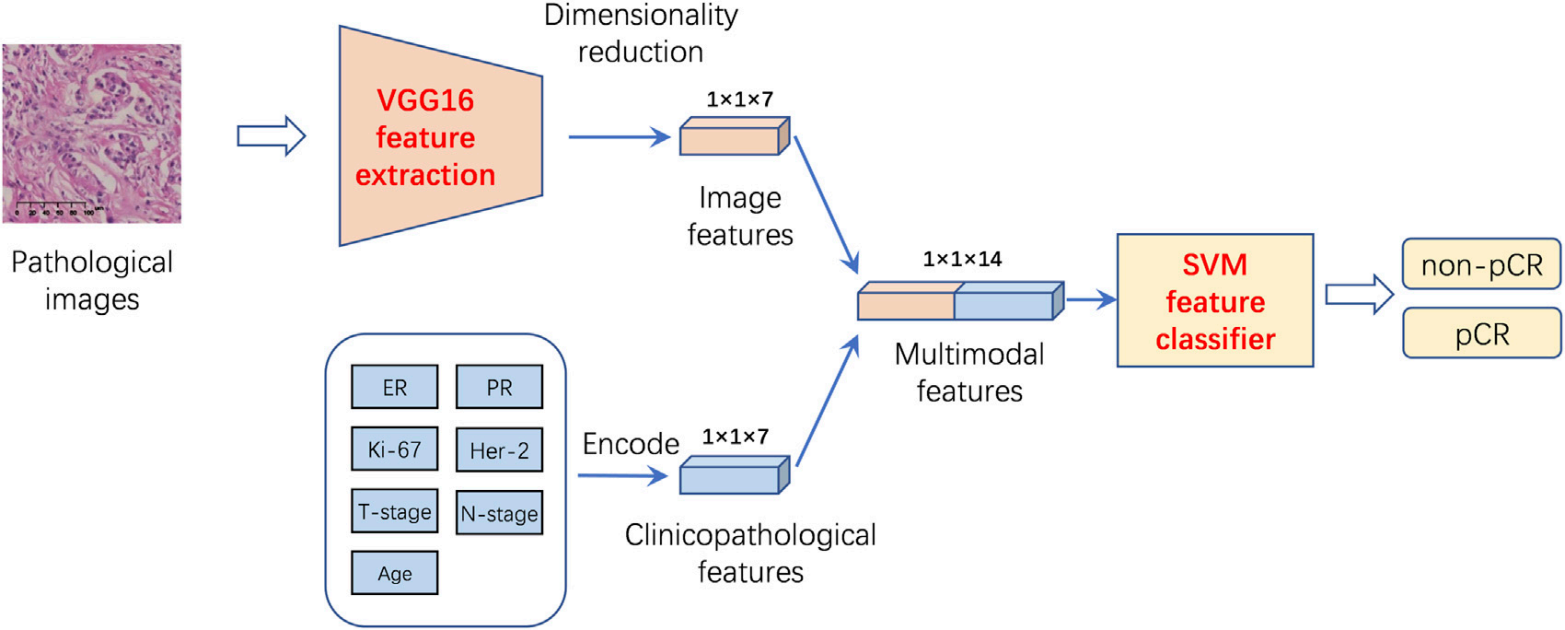
Breast cancer pCR prediction model based on multimodal features and SVM classifier. The VGG16 deep learning model was used to extract features from 20X pathological images. The weights in the VGG16 model, which has been trained on the ImageNet dataset, are transferred into the 13-layer convolutional layer of the feature extraction model. Fine-tune the parameters of the fully-connected layer based on the pCR and non-pCR data. After completing the fine-tuned training, the 13-layer convolutional layer was used as a feature extraction network to obtain a 512-dimensional pathology slice image feature vector. Dimensionality reduction is achieved by a fully connected layer with 7 channels. Then the pathological image features and clinicopathology text features are fused into multimodal features, which were inputted into a radial basis function based support vector machine (SVM) for pCR prediction.

In SMFPM (SVM-based multimodal feature prediction model) Module, firstly, we used the VGG16 transfer learning model for feature extraction on ×20 magnification pathological images. The specific method is as follows: The weights in the VGG16 model, which has been trained on the ImageNet dataset, are transferred into the 13-layer convolutional layer of the feature extraction model. Fine-tune the parameters of the fully connected layer based on the pCR and non-pCR data. After completing the fine-tuned training, the 13-layer convolutional layer was used as a feature extraction network to obtain a 512-dimensional pathology slice image feature vector. Dimensionality reduction is achieved by a fully connected layer with 7 channels. Subsequently, the pathological image features and clinicopathology text features are fused into multimodal features, which are inputted into the SVM classifier. Finally, a multimodal features pCR prediction model (DPCM) was constructed. The specific flowchart is shown in Figure 4. The experimental results show that the combination of pathological image features and text features has better prediction performance than single pathological image features or clinicopathology text features.

### 2.3 Statistical methods

In this study, age at diagnosis was a continuous variable, and other clinicopathological features were categorical variables. Continuous variables were described as medians and interquartile range, and categorical variables were described as percentages. All statistical analyses were two-sided, and *p* values of less than 0.05 indicate statistical significance. Predictive performance was assessed by area under the receiver operating characteristic (ROC) curve (AUC). The accuracy, sensitivity, specificity, and F1 score of the models were calculated.

### 2.4 Software and parameters

#### 2.4.1 WSI acquisition and screenshot software parameters

HE-stained tissue slices from patients enrolled in TC and VC2 were scanned to form WSIs with KF-PRO-020-HI produced by Jiangfeng, which has a spatial resolution of 0.25 MPP and a scan magnification of ×40. HE-stained slices of biopsy tissue from patients enrolled in VC1 were scanned to form WSIs with a Panoramic 250Flash II manufactured by 3DHISTECH, Hungary, with a spatial resolution of 0.25 MPP and a scan magnification of ×40. We browsed WSIs with K-viewer (1.7.0.29) developed by K-Tron International, which supports viewing WSIs in different multiples. Take a screenshot with FSCapture software. Set the screenshot size to 512 × 512 pixels, image resolution to 96DPI, and output to JPG format.

#### 2.4.2 Statistical analyses software and deep learning runtime environment

Statistical analyses were performed in Python 3.8.2. The DL-model and code were implemented based on Pytorch and Python 3.8.2. Deep learning server operation using an i7-11700k processor and an NVIDIA RTX3090 24 GB graphics card. The model is parameter optimized using SGD with a learning rate of 1e-3, a weight decay factor of 5e-4, and a learning momentum of 0.9, with a maximum of 200 training rounds.

## 3 Result

### 3.1 Patient characteristics

The clinicopathological features of the patients are summarized in Table 1. As shown in Table 1, for TC, VC1, and VC2, the median age of patients was 52, 50, and 44.5, respectively. ER positivity rates were 60.1%, 69.2%, and 81.9%, respectively. Her-2 positivity rates were 40.2%, 60.7%, and 33.3%, respectively. The proportion of patients with Ki67 high expression was 77.8%, 88.8%, and 73.6%, respectively. The pCR rates were 28.7%, 36.4%, and 13.9%, respectively.

**TABLE 1:** Clinical characteristics of patients in the training cohort and validation cohorts.

Characteristic	Training cohort	Validation cohort 1	Validation cohort 2
	N = 261	N = 107	N = 72
Age (years) Median (IQR)	52 (46–58)	50 (42.5–56)	44.5 (40.75–52.25)
ER status (%)			
Positive	157 (60.1)	74 (69.2)	59 (81.9)
Negative	104 (39.9)	33 (30.8)	13 (18.1)
PR status (%)			
Positive	125 (47.9)	64 (59.8)	40 (55.6)
Negative	136 (52.1)	43 (40.2)	32 (44.4)
Her-2 status (%)			
Positive	105 (40.2)	65 (60.7)	24 (33.3)
Negative	156 (59.8)	42 (39.3)	48 (66.7)
Ki-67 index (%)			
≥20%	203 (77.8)	95 (88.8)	53 (73.6)
<20%	58 (22.2)	12 (11.2)	19 (26.4)
cT stage (%)			
cT1-T2	104 (39.9)	52 (48.6)	41 (56.9)
cT3-T4	157 (60.1)	55 (51.4)	31 (43.1)
cN stage (%)			
cN0-N1	109 (41.8)	50 (46.7)	21 (29.2)
cN2-N3	152 (58.2)	57 (53.3)	51 (70.8)
NAC efficacy (%)			
pCR	75 (28.7)	39 (36.4)	10 (13.9)
Non-pCR	186 (71.3)	68 (63.6)	62 (86.1)

ER, estrogen receptor; PR, progesterone receptor; Her-2, human epidermal growth factor receptor 2; pCR, pathological complete response.

### 3.2 Performance of different magnification ROIs in each deep learning model in training cohort (TC)

In TC, there are 4 different magnifications (×4, 10X, ×20, ×40) and mix magnifications, each with 5970 ROIs. These ROIs were pre-trained by the ImageNet dataset with transfer learning. The accuracy, sensitivity, specificity, and F1 score of the two benchmark models, VGG16 and ResNet50, in pCR prediction, are shown in Table 2. In both benchmark models, models generated from ×20 magnification pathological images (×20 model) demonstrated the best performance in pCR prediction compared to models generated from images with other magnification. The accuracy, specificity, sensitivity, and F1 scores of the VGG16 model (20X model) in pCR prediction were 0.7487, 0.7294, 0.7642 and 0.7714, respectively. The accuracy, specificity, sensitivity, and F1 scores of the ResNet50 model (20X model) were 0.7173, 0.7284, 0.7264, and 0.7353, respectively. The pCR predictive performance of the VGG, ResNet, ResNeSt, and DenseNet series models using ×20 magnification pathological images from TC are shown in Table 3. As shown in Table 3, in general, the VGG16 model demonstrated the overall best performance, with accuracy, specificity, sensitivity, and F1 score in pCR prediction being 0.7765, 0.7385, 0.7651, and 0.7745, respectively.

**TABLE 2:** Comparison of classification results with different magnification ROIs in training cohort (TC).

Models	Multiples	Accuracy	Specificity	Sensitivity	F1 score
VGG16	4X	0.6178	0.6706	0.5755	0.6456
	10X	0.7016	0.6118	0.7736	0.7421
	20X	0.7487	0.7294	0.7642	0.7714
	40X	0.6387	0.5882	0.6792	0.6761
	MIX	0.6803	0.6752	0.6645	0.7032
ResNet50	4X	0.5916	0.3412	0.7925	0.6829
	10X	0.6754	0.6353	0.7075	0.7075
	**20X**	0.7173	0.7284	0.7264	0.7353
	40X	0.6440	0.5412	0.6792	0.6761
	MIX	0.6705	0.5844	0.7038	0.7057

ROI, regions of interest.

**TABLE 3:** Comparison of the results of 20X ROIs in different classification models in training cohort (TC).

Models	Accuracy	Specificity	Sensitivity	F1 score
VGG16	0.7765	0.7385	0.7651	0.7745
VGG19	0.7472	0.7456	0.7482	0.7503
ResNet50	0.7216	0.7156	0.7265	0.7324
ResNet101	0.7069	0.7264	0.7463	0.7131
ResNest50	0.7364	0.7530	0.7291	0.7382
ResNest101	0.7142	0.7135	0.7160	0.7213
DenseNet121	0.7179	0.6814	0.7177	0.7020
DenseNet161	0.7032	0.6732	0.6761	0.6938

### 3.3 PCR prediction performance of different models

**FIGURE 5:**
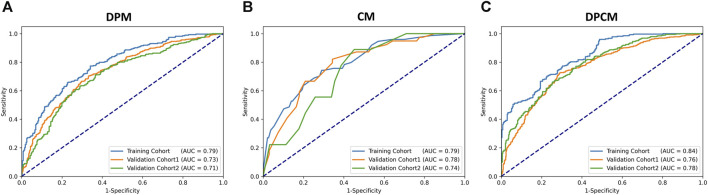
The ROC curve for pCR prediction performance in the **(A)** DPM, **(B)** CM, and **(C)** DPCM among all cohorts. AUC: area under the receiver operating characteristic. CM: clinical model. DPCM: deep learning clinicopathological model. DPM: deep learning pathological model. ROC: receiver operating characteristic.

The AUCs of the DPM in pCR prediction were 0.79, 0.73, and 0.71 in TC, VC1 and VC2, respectively. CM demonstrated similar predictive performance, with AUCs of 0.79, 0.78, and 0.74 in TC, VC1 and VC2, respectively. Notably, the combined model DPCM provided more satisfactory predictive efficacy. In TC and VC2, the AUCs of DPCM were 0.84 and 0.78, respectively, higher than that of the other two models. The predictive performance of the three models is shown in Figure 5 and Table 4.

**TABLE 4:** pCR prediction performance of different models.

Cohort	DPM AUC (95%CI)	CM AUC (95%CI)	DPCM AUC (95%CI)
Training Cohort	0.79 (0.74–0.82)	0.79 (0.73–0.85)	0.84 (0.80–0.87)
Validation Cohort 1	0.73 (0.70–0.76)	0.78 (0.69–0.87)	0.76 (0.74–0.79)
Validation Cohort 2	0.71 (0.67–0.75)	0.74 (0.57–0.87)	0.78 (0.75–0.81)

pCR, pathological complete response; DPM, deep learning pathological model; CM, clinical model; DPCM, deep learning pathological clinical model; AUC, area under the curve; CI, confidence interval.

After calculation, in DPCM, the optimal cutoff for TC is 0.77, and when the optimal cutoff is obtained, the accuracy, specificity, and sensitivity of the model are 0.73, 0.79, and 0.66, respectively. When this cutoff is applied to VC1 and VC2, the accuracy, specificity, and sensitivity of VC1 are 0.71, 0.76, and 0.63, respectively; and that of VC2 is 0.70, 0.75, and 0.62, respectively. The results are shown in Table 5.

**TABLE 5:** pCR prediction performance of DPCM in the optimal cutoff value.

Cohort	Accuracy	Specificity	Sensitivity
Training Cohort	0.73	0.79	0.66
Validation Cohort 1	0.71	0.76	0.63
Validation Cohort 2	0.70	0.75	0.62

pCR, pathological complete response; DPCM, deep learning pathological clinical model.

In addition to comparing AUC results, we added two indices, the net reclassification index (NRI) and the integrated discriminant improvement (IDI), to further evaluate the model performance of DPCM and DPM. Compared with DPM, the NRI values of TC, VC1, and VC2 in DPCM are 0.054, 0.019, and 0.061, respectively, and the IDI values are 0.042, 0.014, and 0.057, respectively, and the computational results show that the predictive effectiveness of DPCM has a small improvement compared with DPM.

### 3.4 PCR prediction performance of DPM and DPCM in different molecular subtypes in validation cohorts (VCs)

**FIGURE 6:**
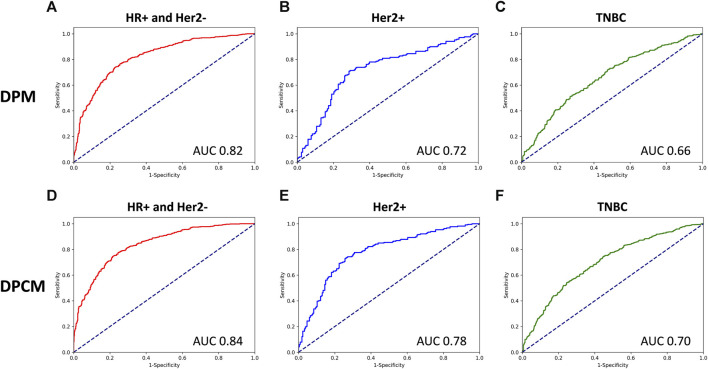
The ROC curve for pCR prediction performance of DPM and DPCM in different molecular subtypes in validation cohorts. The ROC curve for pCR prediction performance of DPM in **(A)**. HR+ and Her2-, **(B)**. Her2+, **(C)**. TNBC. The ROC curve for pCR prediction performance of DPCM in **(D)**. HR+ and Her2-, **(E)**. Her2+, **(F)**. TNBC. ROC: receiver operating characteristic. AUC: area under the receiver operating characteristic. DPM: deep learning pathological model. DPCM: deep learning clinicopathological model. HR: hormone receptor. Her2: human epidermal growth factor receptor 2. TNBC: triple-negative breast cancer.

The AUCs of the DPM in HR (hormone receptor) positive and Her2 negative, Her2 overexpressing, and TNBC (triple-negative breast cancer) were 0.82, 0.72, and 0.66, respectively. The AUCs of the DPCM in HR positive and Her2 negative, Her2 overexpressing, and TNBC were 0.84, 0.78, and 0.70, respectively. The results are shown in Figure 6 and Table 6.

**TABLE 6:** pCR prediction performance of DPM and DPCM in different molecular subtypes in validation cohorts.

Subtypes	DPM AUC	DPCM AUC
HR+ and Her2-	0.82	0.84
Her2+	0.72	0.78
TNBC	0.66	0.70

pCR, pathological complete response; AUC, area under the receiver operating characteristic; DPM, deep learning pathological model; DPCM, deep learning clinicopathological model; HR, hormone receptor; Her2 human epidermal growth factor receptor 2; TNBC, triple-negative breast cancer.

## 4 Discussion

In this study, we found that pathological images of HE-stained slices of pre-NAC biopsy tissues could be used for building models to predict the treatment efficacy of NAC in breast cancer. VGG16 model generated from ROIs of ×20 magnification demonstrated the best predictive performance compared with models generated from ROIs of other magnification. The combined model had superior predictive efficacy than the deep learning model or the clinicopathological model.

HE-staining pathological images contain a large amount of information about tumor epithelial cells and stromal. Prediction of anti-tumor treatment efficacy and prognosis using deep learning features provided by pathological images has been attempted in liver cancer (Saillard et al., 2020), malignant mesothelioma (Courtiol et al., 2019), and rectal cancer (Shao et al., 2020). However, limited similar studies have been performed on breast cancer. This study showed that deep learning features from pathological images were predictive of NAC efficacy in breast cancer. Our results show that the model included only DL-features of pathological images had an AUC of 0.79 in the TC and 0.73 and 0.71 in the two external VCs, respectively. These results are similar to results from a recent study, in which the AUC was 0.72 in predicting pCR using a DP features-based model (Li et al., 2022a).

In this study, we used screenshot software to select the ROIs from HE-staining pathological images at different magnifications (×4, 10X, 20X, 40x), all with a screenshot size of 512 × 512 pixels. The results showed that the DL-features from ×20 magnification images achieved the best performance in predicting pCR. The lower predictive efficacy of pathology images under ×4 and ×10 magnification field of view may be because the screenshot software used in this study limited the pixel size of ROIs. Although the ROIs selected at ×4 and ×10 magnification covered more tumor cells and stromal, the ROIs were not clear enough for feature extraction. ×40 magnification images provide better observation of tumor cell morphology and even nucleus division, but the ×512512 pixel size image contains fewer tumor cells, which is not conducive to the observation of tumor cell arrangement. In contrast, ×20 magnification pathological images include more tumor cells and stromal at the same figure, providing more adequate information on tumor morphological features and sTILs. A study by Dmitrii Bychkov et al. showed that the tumor morphological features can be used to predict the efficacy of NAC in Her-2 overexpressing breast cancer, also. (Bychkov et al., 2021). As for the predictive value of sTILs on the treatment efficacy of NAC in breast cancer, it also has been confirmed in many studies (Hwang et al., 2019; Ochi et al., 2019; Sun et al., 2021). Therefore, in this study, ×20 magnification pathological images had the best pCR prediction efficacy is possessed interpretability.

In this study, we used multiple deep learning models for feature extraction and found that the classification metrics of the VGG model are significantly better than ResNet, ResNest, and DenseNet. This is due to the fact that the pre-trained models are generally obtained by training with color natural image data from ImageNet. Since color natural images are complex and have higher dimensional features, the use of deep convolutional neural networks such as ResNet, ResNest, and DenseNet to extract features, better results can be obtained. Whereas compared to color natural images, pathology images are simpler and do not have very complex features, extracting features using the deeper number of layers and complex structure of pre-trained models such as ResNet, ResNest, and DenseNet will result in overfitting of features. On the contrary VGG model with a simple structure and low number of network layers is suitable for feature extraction from pathology slice images (Chen et al., 2022). Therefore, VGG16 and VGG19 outperform ResNet, ResNest, and DenseNet in the problem of classification of pathology images. And VGG16 has fewer layers compared to VGG19, so VGG16 has better classification results. In this study, the accuracy, specificity, and sensitivity of the model were 0.73, 0.79, and 0.66, respectively, when VGG16 achieved optimal cutoff in DPCM. So far, to the best of our knowledge, no attempt has been made in other studies to evaluate the predictive performance of multiple models in a single study. Therefore, the results from this study are more reliable.

In addition to utilizing information from pathological images, it is also common to use important clinicopathological features, such as T-stage, N-stage, ER, PR, Her-2, Ki-67, and molecular typing, to build models for predicting pCR. In a study by Qian et al., the clinical model, including pre-NAC T-stage, ER, Her2, and Ki-67, demonstrated good performance in predicting pCR in breast cancer with an AUC of 0.79 in TC (Qian et al., 2022). This result is close to our CM. However, it needs to be discussed that after fusing pathological image features and clinicopathological features, the predictive efficacy of DPCM was improved in TC and VC2 compared to both DPM and CM. On the contrary, in VC1, the predictive efficacy of DPCM was not as good as CM. We analyze that this may be due to the following two reasons. 1. There are only seven clinicopathological features included in this study, and they are affected by the uneven enrollment ratio of different subtypes in different centers, which may result in large fluctuations in the prediction efficacy of CM in different centers. 2. Pathological image and clinicopathological features provide different amounts of effective information. Compared to clinicopathological features, deep learning extracts features from pathological images with higher dimensionality and more effective information, which plays a more important role in the robustness and accuracy of the model.

Using the predictive model, we can obtain a predicted probability of pCR after NAC for breast cancer, and there is a certain difference between this probability and the pathological results of undergoing surgery after NAC, i.e., the gold standard, which will lead to a certain degree of uncertainty when the predictive model is applied. The estimation of model uncertainty depends on many factors. Primarily, pathologic images and clinicopathologic parameters alone provide only limited information in predicting pCR. If multimodal data are added, such as ultrasound (Cui et al., 2021), CT (Moghadas-Dastjerdi et al., 2021), MRI (Huang et al., 2023; Shi et al., 2023), PET-CT (Yang et al., 2022) examination data, or even genetic testing data, the predictive efficacy of the model can be further improved and the uncertainty of model application can be reduced. Secondly, the number of enrolled cases and the number of clinicopathologic features also affect the uncertainty of model application. In recent relative research, many techniques such as radiomics or genomics have also been explored for NAC efficacy prediction. For example, multi-landscape histology techniques were used to build an NAC efficacy prediction model by whole-genome sequencing of puncture tissues from 168 breast cancers. External validation of the model in 75 patients showed good predictive performance with an AUC of 0.87 (Sammut et al., 2022). This approach takes full advantage of the information provided by the tumor ecosystem for efficacy prediction. However, whole genome sequencing is very expensive and therefore difficult to apply universally in clinical practice. In another study, the combination of clinicopathological features and MRI signatures before and after NAC has also been used to predict NAC efficacy with an AUC of 0.90 (Kim and Cho, 2021). Despite the good performance in treatment efficacy prediction, these models have drawbacks, such as the need for repeated examinations at different time points during the treatment course. This will result in more medical costs and cannot truly achieve early prediction. In contrast, pretreatment tissue biopsy is a routine procedure in the diagnosis and treatment of breast cancer, the model built in this study does not add additional workload and cost and holds the promise for early treatment efficacy. Therefore, it is still worthwhile to continue exploring how to balance the uncertainty, practicality, and economics of predictive modeling applications.

Our study has some limitations. First, breast cancer is highly heterogeneous. The pathological information provided by core needle biopsy is not fully representative of the entire tumor. Nevertheless, the overall predictive accuracy of our DPM model is quite good as demonstrated in this study, and will even be better when combined with the clinicopathological model. Second, this is a retrospective study with a small patient size. The uneven distribution of molecular subtypes in the three centers may have affected the results. From the above results, it can be seen that the efficacy of DPM and DPCM in predicting pCR was better than the other two subtypes in the HR + Her2-subtype. We analyzed that it might be due to the difference in the number of cases enrolled in the three subtypes when the model training was performed in this study, with the HR + Her2-subtype enrolled in the largest number of cases and the TNBC subtype enrolled in the smallest number of cases. In.

TC, the proportion of patients with HR + Her2-, Her2 overexpressing, and TNBC subtypes was 46.6%, 36.1%, and 17.3%, respectively. The information extracted from the cases of different subtypes during the training of the model varied, thus leading to different predictive efficacy of the model when tested on cases of different subtypes. In the future, we will analyze the predictive efficacy of the model in cases of different subtypes again when more cases are collected and the number of cases enrolled in different subtypes is more balanced. Third, the method of selecting ROIs in this study is not fully automated and may lead to subjective differences. However, this study has a clear definition of how to select ROIs, and the entire process of selecting ROIs involved a breast surgeon and a pathologist to ensure that the selection was done strictly according to the definition. In addition, we selected 5 ROIs from each WSI, which allows ROIs to be better representative of WSIs and minimizes subjective differences in manual screening. In similar studies published so far, although ROIs can be selected automatically, the preliminary stage of model building requires manual patch type delineation or cell labeling, both of which are labor-intensive and inherently subjective differences. For example, in the study of Li et al. (Li et al., 2022a), a large number of patches needed to be manually classified in the preliminary stage to train the model and construct the patch classifier, which was used to predict pCR with an AUC of 0.72. In the study of Li et al. (Li et al., 2021), the tumor epithelial region needed to be manually labeled in the preliminary stage to train the model, which was used to predict pCR with an AUC of 0.847. From the results of this study, the AUC of the training group was 0.79, and the prediction efficacy was close to these two studies. Therefore, the use of non-automatic selection of ROIs in this study did not have a significant impact on the predictive performance of the model. In the future, we will conduct more in-depth research on how to select ROIs accurately and efficiently.

## 5 Conclusion

Our study demonstrates that DL-features from HE-stained slices of pre-NAC biopsy tissues could potentially predict pCR in patients with breast cancer. Combination with clinicopathological features will further improve the predictive efficacy.

## Data Availability

The raw data supporting the conclusion of this article will be made available by the authors, without undue reservation.
